# Distinct and combined responses to environmental geometry and features in a working-memory reorientation task in rats and chicks

**DOI:** 10.1038/s41598-020-64366-w

**Published:** 2020-05-05

**Authors:** Sang Ah Lee, Joseph M. Austen, Valeria Anna Sovrano, Giorgio Vallortigara, Anthony McGregor, Colin Lever

**Affiliations:** 1 Department of Bio and Brain Engineering, Korea Advanced Institute of Science and Technology, Daejeon, Korea; 2 Department of Psychology, Durham University, Durham, UK; 3 Centre for Mind/Brain Sciences, University of Trento, Rovereto, Italy; 4 Department of Psychology and Cognitive Science, University of Trento, Rovereto, Italy

**Keywords:** Animal behaviour, Psychology, Spatial memory

## Abstract

The original provocative formulation of the ‘geometric module’ hypothesis was based on a working-memory task in rats which suggested that spontaneous reorientation behavior is based solely on the environmental geometry and is impervious to featural cues. Here, we retested that claim by returning to a spontaneous navigation task with rats and domestic chicks, using a single prominent featural cue (a striped wall) within a rectangular arena. Experiments 1 and 2 tested the influence of geometry and features separately. In Experiment 1, we found that both rats and chicks used environmental geometry to compute locations in a plain rectangular arena. In Experiment 2, while chicks failed to spontaneously use a striped wall in a square arena, rats showed a modest influence of the featural cue as a local marker to the goal. The critical third experiment tested the striped wall inside the rectangular arena. We found that although chicks solely relied on geometry, rats navigated based on both environmental geometry and the featural cue. While our findings with rats are contrary to classic claims of an impervious geometric module, they are consistent with the hypothesis that navigation by boundaries and features may involve distinct underlying cognitive computations. We conclude by discussing the similarities and differences in feature-use across tasks and species.

## Introduction

Thirty-five years ago, a remarkable set of experiments showed that disoriented rats relied on the shape of the test arena to return to a food source, while ignoring landmarks or other “featural” cues such as patterned panels and distinctive odors^1,2^. These results motivated the claim that reorientation is based exclusively on environmental geometry and is independent from the control of other featural cues^3^. Because the possibility of a *purely geometric module* provided new and important insights into the cognitive processes underlying spatial behavior, many studies on reorientation have followed in a wide variety of species, including humans^4–6^.

Across this large literature, however, results have been inconsistent and interpretations keenly contested. One fundamental reason for the debate is that there are no clear standards for what should be taken as evidence for or against modularity. In earlier works^2,7^ the *failure* to use featural cues in conjunction with geometry to guide spatial behavior was presented as the strongest demonstration of encapsulation^8^. Other experiments showed that although animals and human children can learn to use features to confine their searches to the goal location, their success often relied only on those cues that were placed directly near the goal; that is, when local features at the goal were removed, animals no longer showed a preference for the goal based on spatial relationships with respect to the remaining featural cues (e.g., rats^2^; chicks^9^; children^10^). Arguments for or against modularity have often been made based on interactions (or lack thereof) between geometry and features^11^. In nonhuman animals, such studies have mainly adhered to reference-memory paradigms, often involving extensive training and reinforcement of choice behavior at a single goal location^4^. A wide range of studies have demonstrated the associative effects of feature learning on geometry-based spatial mapping in these tasks; overshadowing^12,13^, blocking^14,15^, learned attentional changes^16,17^, within-compound associations between geometry and features^18–20^, conditioned inhibition^21^ and potentiation of geometry learning by featural cues^15,21–24^ have all shown that non-geometric features can interact with the progression of geometry learning. However, successful use of featural cues in these paradigms has been argued to reflect the engagement of associative processes that do not recruit the specialized cognitive computations engaged in the use of geometric cues following disorientation. That is, reference memory tasks such as those discussed above recruit general learning systems parallel to the specialized computations employed in the use of boundary geometry. Reference memory tasks, therefore, may mask detection of the modular nature of boundary-based spatial mapping by recruitment of associative processes. For such reasons, some researchers emphasize the importance of using a working memory task in observing distinctions between geometry and feature use^25,26^. It is important to note, however, that even in working memory or spontaneous tasks, researchers have found varying degrees of feature use depending on the task (e.g., aversive vs. appetitive tasks in rats^27^; verbal cuing in children^28^) or environment (e.g., large vs. small environments^29,30^, circular/octagonal environments^10,31^). Because alternative explanations for the observation of interactions between geometry and features are available, it seems that evidence for or against independent computation of geometric and featural cues rests not on *whether* a cue influences behavior, but on *how* it influences behavior^25^. Such differences in function may additionally lead to hypotheses about both the cognitive computations and neural representations underlying spatial navigation.

A recent study with fish provides a clear demonstration of the importance of both the task and the available environmental cues in disoriented spatial behavior^32,33^. The two types of tasks (working memory and reference memory) were tested across three conditions: geometry-only, features-only, and a combination of geometry and features. As in previous studies, environmental geometry immediately guided navigation in both tasks, and features were reliably learned in the reference memory task (although they learned local features significantly faster than the distal ones). In contrast, in the working memory type task, visual landmarks acquired perceptive salience and attracted the fish but without serving as a spatial landmark when they were located far from the target location. Interestingly, however, when provided simultaneously with environmental geometry, the featural cues – whether those cues were distinctive corner panels or a single uniquely-colored wall^33^ - led the fish to limit their choices to the correct corner significantly more often, even in the absence of reinforced training.

Similarly, behavioral studies with mice have independently tested the use of geometry and landmarks in both a working memory task and a reference memory task^34^. While mice reoriented by geometry from the very start in both tasks, a featural cue (i.e. striped wall) was successfully used only as a local marker (but without any sense of left versus right) in the working-memory task. With reinforcement in the reference-memory task, however, mice became increasingly accurate in identifying the one target corner. Interestingly, genetically modified mouse models of neurological disorders showed selective learning of features and geometry, depending on their hippocampal dysfunction or attentional deficit^35^. In those studies, however, there was no simultaneous testing of geometry with a featural cue, making it unclear whether the presence of a geometric structure enhances feature-use in rodents the way it did in fish, particularly in the spontaneous “working-memory” task.

The present study tests the generalizability of such findings across different species of animals through a working-memory test of rats and domestic chicks, presenting geometry and features in isolation (Experiments 1 and 2) and in conjunction (Experiment 3). The task involved observing disoriented animals’ corner preferences (in the absence of reward) after having previously found food in one corner of an arena. Experiment 1 examined the use of rectangular environmental geometry using a uniformly black arena; Experiment 2 examined feature-use in a square arena with a single striped wall; Experiment 3 tested the simultaneous use of geometry and features using a rectangular arena with a single striped wall. Given the potential effects of arena size, we kept our rectangular arena’s dimensions the same as the original Cheng study^2^. However, instead of corner panels, we chose to implement a single striped wall, in accord with the above studies on mice and based on single landmark control over head direction cell responses^36,37^, using a spatial frequency based on considerations of rodent visual acuity^38,39, which is lower than that of chicks40^. Different subjects were used for each experiment. These arenas were set in well-controlled testing rooms designed to offer no cues to orientation other than those in the arenas themselves.

## Results

### Experiment 1: Geometry

**Figure 1:**
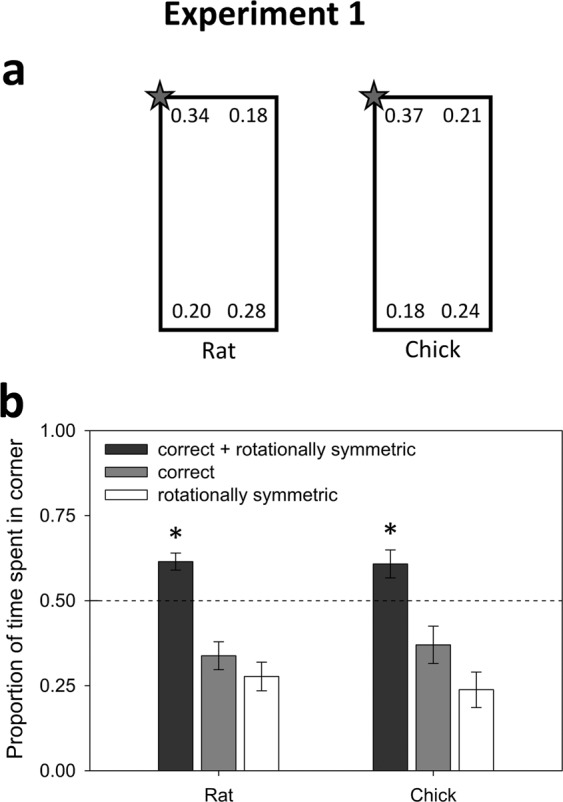
Results of Experiment 1. (**a**) Corner preferences by rats and chicks, as measured by the proportion of time spent in each corner. The correct corner is denoted with a star. Because the target corner was varied across trials, the data have been rotated prior to averaging and are displayed in this rotated form. (**b**) Both rats and chicks were guided by the rectangular layout of the arena, as measured by the proportion of time spent in the correct and featurally symmetric corners. Error bars show the standard error of the mean; asterisks denote significant t-tests against a 0.5 chance level with p < 0.05. The preference for the correct corner was not statistically greater than the rotationally symmetric corner for either rats and chicks.

Experiment 1 tested the use of environmental geometry using a uniformly black-colored rectangular arena (see Fig. 1a). Due to rotational symmetry, searches to both the correct corner and the diametrically opposite corner were recorded as being geometrically correct. Importantly, if the subject is successfully disoriented, and no uncontrolled odor cues mark the correct location, the correct and geometrically identical corners should not be distinguishable. Eleven rats (mean = 5.7 trials) and ten chicks (mean = 4.4 trials) were observed. Results from one other chick were omitted due to signs of distress.

#### Rats

The proportion of time spent at the geometrically correct corners was significantly greater than a chance level of 0.5 (t(10) = 4.59, p = 0.001, Cohen’s *d* = 1.38, see Fig. 1b). Importantly, there was no sign of preference for the correct corner over its geometric equivalent (t < 1, p > 0.25, Cohen’s *d* = 0.23), indicating an effective disorientation procedure with no lingering reward-odor cues in our working-memory paradigm.

#### Chicks

The proportion of time spent at the geometrically correct corners was significantly greater than a chance level of 0.5 (t(9) = 2.64, p = 0.03, Cohen’s *d* = 0.95, Fig. 1). Again, importantly, there was no preference for the correct corner over its geometric equivalent (t(9) = 1.34, p = 0.21, Cohen’s *d* = 0.19).

Both rats and chicks searched in accord with boundary geometry. This result is consistent with past findings in rats^2^ and in chicks^41^. The equal preference between the correct corner and the geometrically equivalent corner indicates an effective disorientation procedure and provides us with an internal control that ensures the absence of cues that might make the correct corner uniquely identifiable.

### Experiment 2: Feature

**Figure 2:**
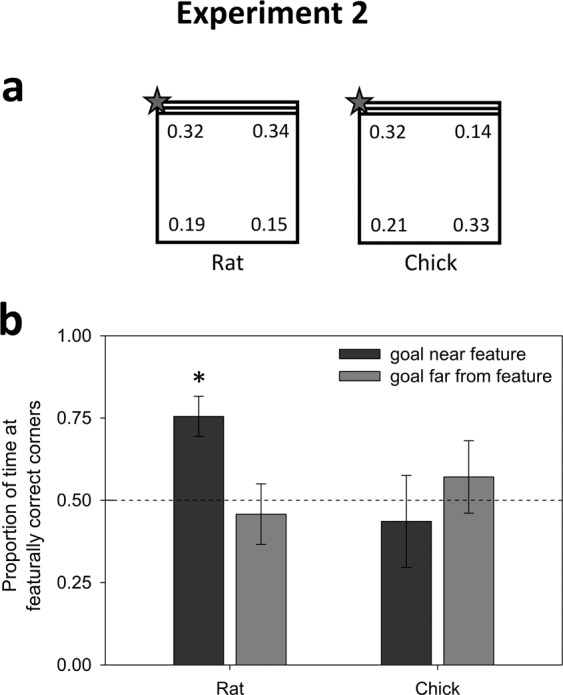
Results of Experiment 2. (**a**) Corner preferences by rats and chicks, as measured by the proportion of time spent in each corner. The correct corner is denoted with a star. Because the target corner was varied across trials, the data have been rotated prior to averaging and are displayed in this rotated form. Rats preferred the correct and featurally symmetric corners (i.e., correctly matching the presence/absence of the striped wall with the target) over the other two, while the chicks did not use the striped wall to guide their behavior. (**b**) The rats’ use of the striped feature as a cue (the proportion of time spent in the correct and featurally symmetric corners) was limited to the trials in which the goal was near the stripes (rather than the all-black side of the arena). The asterisk denotes a significant t-test against a 0.5 chance level with p < 0.05. Chicks did not use the striped feature, even when it served as a local cue to location.

Experiment 2 tested reorientation by a feature in a square arena with a single striped wall (see Fig. 2a). If animals distinguished all four corners with respect to the feature (i.e., relative directions or “sense”), there should be a preference for the correct corner over all others. A more limited use of the feature would be if animals used the striped wall as a direct local feature, without using it to compute sense relations. In this case, for instance, we should detect a preference for the two striped corners when the goal was at a striped corner. Nine rats (mean = 5 trials) and eight chicks (mean = 5 trials) were observed.

#### Rats

The proportion of time spent at the correct corner was not significantly different from a chance value of 0.25 (t(8) = 1.64, p = 0.14, Cohen’s *d* = 0.55). This indicates a failure to compute ‘sense’ (i.e., left/right-ness) from the featural cue. Nevertheless, there was a clear statistical preference for featurally correct over featurally incorrect corners (t(9) = 3.27, p = 0.01, Cohen’s *d* = 1.09, see Fig. 2b). Interestingly, however, further inspection showed that this effect was driven by trials in which the goal was near the striped wall: when the goal was at a striped corner, rats spent significantly more time at the two striped corners (72%: t(8) = 4.21, p = 0.003, Cohen’s *d* = 1.40) but when the goal was at an all-black corner, rats did not exhibit a preference for the black corners (46%: t < 1, p > 0.25, Cohen’s *d* = 0.16). In both cases, rats did not distinguish the correct corner from the symmetric corner (t’s < 1, p’s> 0.25), indicating a complete failure to use the striped wall to compute directional relationship between locations. In summary, Experiment 2’s results indicated a clear, if relatively modest, influence of the featural cue; the rats could not use it to extract ‘sense’ but leveraged a ‘goal proximity’ benefit from it, consistent with use of the feature as a beacon.

#### Chicks

The proportion of time spent at the correct corner was not statistically different from chance of 0.25 (t < 1, p > 0.25, Cohen’s *d* = 0.27); whether the corner was striped or black did not make a significant difference in performance (t(7) = 1.03, p = 0.34, Cohen’s *d* = 0.39, Fig. 2). Moreover, chicks did not respond to the striped feature as a cue to distinguish between the two striped corners and the two black corners, whether the goal was at a striped or all-black corner (all t’s <1, p’s> 0.25). Although this null effect should not be over-interpreted, the fact that chicks were not significantly influence by the featural cue cannot be attributed to an inability to perceive it, given their clear success in learning to associate it with a goal when provided with reinforcement (see Exp. 4 in Supplementary Materials); in the free-exploration probe trial, chicks (n = 6) spent 91.9% of their time on the side of the arena with the stripes (t(5) = 8.06, p < 0.001).

### Experiment 3: Geometry and Feature

**Figure 3:**
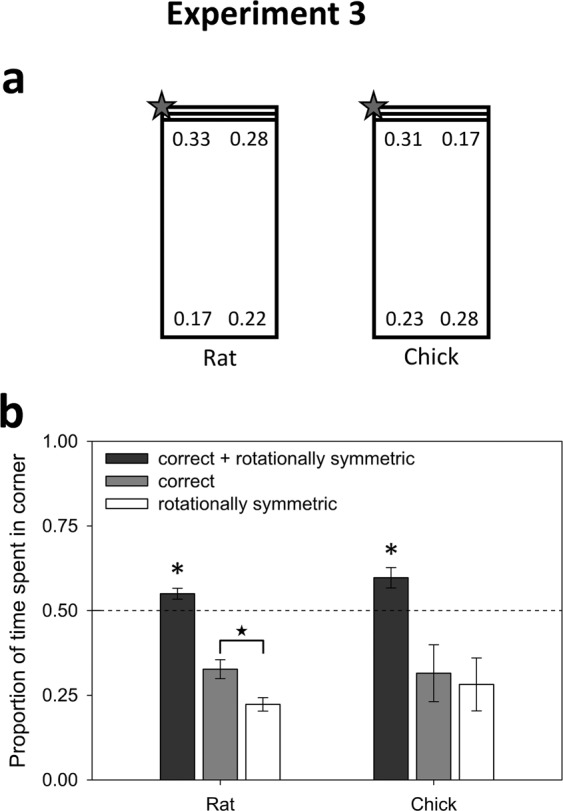
Results of Experiment 3. (**a**) Corner preferences by rats and chicks, as measured by the proportion of time spent in each corner. The correct corner is denoted with a star. Because the target corner was varied across trials, the data have been rotated prior to averaging and are displayed in this rotated form. (**b**) The rats use the striped wall to discriminate between the correct corner and the rotationally symmetric corner, while the chicks are only guided by boundary geometry. Asterisks denote significant t-tests of geometry (correct + rotationally symmetric corners) against a 0.5 chance level with p < 0.05. Star denotes the paired t-test between the correct and rotationally symmetric corners, with p = 0.05.

Experiment 1 demonstrated that both chicks and rats spontaneously used boundary geometry to compute spatial locations. Experiment 2 showed that although rats did not compute relative positions with respect to the feature, they used it as a local feature to guide search. In contrast, the chicks showed no sign of feature-use with respect to the striped wall and explored the corners at random. If both geometry and features are present simultaneously, will one of those cues dominate over the other? Or, will both of them influence behavior? Experiment 3 tested the combined use of environmental geometry and a feature when both were present, in a rectangular arena with one striped wall (see Fig. 3a). Nine rats (mean = 5.7 trials) and eight chicks (mean = 5.8 trials) were observed.

#### Rats

As in Experiment 1, the combined proportion of time spent at the two geometrically correct corners was significantly higher than a chance level of 0.5, (t(8) = 3.05, p = 0.02, Cohen’s *d* = 1.02, see Fig. 3b). However here, unlike Experiment 1, rats tended to prefer the correct corner over the geometrically identical diagonal corner (t(8) = 2.27, p = 0.05, Cohen’s *d* = 0.76). There was no significant difference in accuracy between goals at the striped and all-black corners (t(8) = 1.39, p = 0.20). In summary, contrary to some interpretations of the original geometric module hypothesis, search time was *immediately* guided by the use of both geometric and featural information. Interestingly, use of both sets of information occurred at the level of individual rats: e.g., the majority of the nine rats showed a pattern whereby search at the correct corner was at least 12% higher than at the rotationally equivalent corner *and* at least 9% higher than at the featurally similar corner (fifth-ranked rat scored: 36% at correct corner, 24% at rotationally equivalent corner, 27% at featurally similar corner, 13% at error corner). In fact, the group average of 17% at the error corner was significantly lower than chance (t(8) = −3.11, p = 0.015)

#### Chicks

Similarly to the rats, the combined proportion of time spent at the correct corner and its geometric equivalent was significantly higher than a chance level of 0.5 (t(7) = 3.19, p = 0.02, Cohen’s *d* = 1.13, Fig. 3); but, in contrast to the rats, there was no discrimination between the correct corner and its geometric equivalent (t < 1, p > 0.25). The proportion of time spent at the correct corner was not significantly different from chance (t < 1, p > 0.25, Cohen’s *d* = 0.27)).

In summary, rats were able to use both environmental geometry and a feature to guide search at the correct goal, while chicks reoriented only on the basis of environmental geometry, despite their tendency to rely on visual features when trained to do so^9^ (see Exp. 4, Supplementary Materials).

## Discussion

The present experiments shed new light on the decades-old debate over the geometric module by accomplishing the following: First, Experiment 1 replicated the main finding that environmental geometry guides spatial navigation in both mammals and birds in the absence of path integration (or positional tracking). Rats and chicks spontaneously used geometry in a working-memory task with varied goal locations and did not need repeated training at one rewarded location to compute spatial relationships among the arena boundaries. This is consistent with the wealth of existing studies demonstrating that spatial mapping relies on a neurocognitive representation of boundary geometry^25,26,42–45^.

Second, Experiments 2 and 3 show, for the first time, that rats can use a featural cue in spatial reorientation. Although chicks did not seem to do so in the present experiment, rats successfully leveraged a featural cue to guide their search in a working-memory task, which, importantly like Cheng’s, tapped ‘spontaneous’ behavior without requiring extensive training. In Experiment 3, they jointly (in one trial) used two sets of information to guide their behavior, one based on environmental geometry, the other based on the featural cue.

These findings clearly weaken the empirical basis underlying a ‘strong’ version of modularity that is deterministic at the level of behavior (i.e. that the output of a geometric module assumed control over behavior without the influence of other processes such as landmark-use). In the critical Experiment 3, the effect size of greater search in the correct than rotationally equivalent corner is Cohen’s d = 0.76, which is far from negligible. In other words, disoriented animals are not limited *only* to environmental geometry in guiding initial search under disorientation. Overall, comparison of effect sizes in our findings is consistent with a greater influence of environmental geometry over features in reorientation, but it is clear that rats can also take features into consideration when performing such tasks. Nevertheless, the *way* in which the feature was used in this working-memory task was limited to a local-marker of the goal (apparent in their preference of locations based on the presence of the striped cue), unlike the relative spatial relationships that were computed with respect to the environmental geometry. This is in line with past findings of disoriented spatial behavior in mice, fish, as well as human children, using features and geometry in isolation^10,25,32–35^.

The results taken together describe an underlying spatial representation of environmental geometry which, in the absence of repeated reinforcement, operates alongside a separate feature-detector with an associative bias (direct-marking)^25,33,42^. This suggests that while the computations involved in the use of geometry and features are different, the output of a single behavioral choice (i.e., in Exp. 3) may involve an adaptive weighting of cues according to properties such as salience and experienced validity^4,46^. However, the patterns of behavior we observed here (and in the studies mentioned above) directly contradict a global image-matching strategy^38^, which would allow animals to distinguish between the two striped corners.

What could explain the rats’ use of the feature as a local cue, given the past findings of failure and the failure on the part of the chicks? First, it may be important to point out that there were several differences between the original methods from Cheng’s^2^ study and ours. First, we used a different strain of rats (Lister Hooded) from those tested in Cheng’s study (Sprague Dawley), although both studies used male rats. Lister Hooded rats have better vision than Sprague Dawleys, but not all the featural cues in Cheng’s study were visual. More importantly, when designing our apparatus, we chose to keep the same size apparatus as Cheng’s but to simplify the featural cue, based upon knowledge of cues used to orient spatial cells^36,37^. Instead of having multiple visual cues and odors, which might introduce issues related to multiple cue discrimination and recognition, we chose a simple, prominent striped pattern with clearly visible contrast edges, at a spatial frequency that rats were sure to perceive, perhaps even more easily than a uniformly white wall^38,40^. We also chose to limit our target locations to corner feeders in an empty arena (that could be cleaned to get rid of odor cues), rather than having the targets be anywhere in a sandbox and allowing rats to dig for the reward. The clear visibility of the feature within our well-lit room and its salience against an otherwise black arena, along with the clear distinction between possible choices of feeders near and far from the feature, may have prompted rats in our study to use the featural cue.

Along the same logic, it is possible that our across-species standardization of the striped cue may have favored rats over chicks, given chicks’ innate preference for smaller visual features^47^. We note, however, that the chicks used that very same cue quite proficiently in a reinforced reference-memory task (Exp. 4, Supplementary materials). Another possibility is that, given the difference in their body size, by using the same-sized apparatus for both chicks and rats we may have inadvertently tested the chicks in comparatively larger environments. However, according to previous studies, larger environments *favor* feature-use^30,31^, which makes this an unlikely explanation for the species differences in this task. One other possibility is that our task protocol in having the rats sample the target location twice (see Methods) provided them with a better representation of the environment. However, even if that were somehow true, it still would not be able to explain the fact that the chicks and rats performed so similarly in their use of boundary geometry (61% vs. 62% geometrically correct preference in Exp. 1), yet so differently in their use of the feature (46% vs. 68% featurally correct preference in Exp. 2).

Study after study, the variability in performance across tasks and species is with respect to feature-use (or the competition between features pitted against geometry), not about the use of geometry itself. Perhaps the crucial point is that variation across species, whether it is due to differences in their perceptual system or other ecological factors^4^, affects the representation of featural landmarks to a greater extent than the representation of environmental structure.

For the past few decades, the geometric module hypothesis has provided a powerful theoretical framework for understanding the central role of environmental boundaries in navigation and spatial mapping. During that time, spatial navigation research has witnessed tremendous progress and a wealth of scientific knowledge that is likely unparalleled in any other area of cognition research, and the study of environmental boundaries and landmarks in spatial representation and behavior has made a significant contribution to that end. Behavioral evidence in a wide range of animal species suggests that computations of environmental geometry involve representations of distance and directions with respect to three-dimensional boundary layouts^48,49^. Evidence from human neuroimaging studies suggests that learning locations with respect to boundaries and landmarks preferentially recruits the hippocampus and dorsal striatum, respectively^50^; a similar distinction has been found in the avian^51^ and rodent brains^52–54^. At the level of single neurons, geometry-based navigation may be supported by boundary-coding neurons such as boundary vector cells in the hippocampal formation^55–57^. Prior to their discovery^42^, boundary vector cells were hypothesized as a major input to hippocampal place cells, which in turn form allocentric “maps” of the surrounding environment in a manner sensitive to environmental geometry^58–60^. Direct visualization of neurons responding to a single-shot experience of environmental boundary transformations in young domestic chicks^61^ and intracranial recordings of boundary-specific increases in theta power from human epilepsy patients performing a computer-based navigation task^62^ suggest that such neural representation of boundaries may be a commonly shared underlying neural correlate of boundary-based navigation. Arguably, a vital challenge now is to combine investigation of these and other types of hippocampal spatial neurons^36^ with reorientation behavior^43,44^ across various species.

Thirty-five years after Ken Cheng’s^2^ formulation of the geometric module, we find ourselves in a new era of research on spatial cognition. We envisage thirty more fruitful years of research to successfully integrate behavioral and neural evidence on the representations of boundaries and features, to provide deeper insight as to how and at what level such representations interact, and to better characterize the principles that are shared and distinct across vertebrate species that ultimately give rise to behavior.

## Methods

### Subjects

#### Rats

Twenty-nine adult male Lister Hooded rats (*Rattus norvegicus*, Harlan Olac, Bicester, England) were housed in groups of five with continuous access to water. They were held on a 12-hr light/dark cycle; testing occurred during the light phase. Rats were on a restricted diet of 15 g of food per animal, beginning two days prior to testing. No animals dropped below 95% of free-feeding weight.

#### Chicks

Twenty-six newly-hatched male chicks (*Gallus gallus domesticus*, Agricola Berica, Vicenza, Italy) were singly housed, with a 13-hr/11-hr light/dark cycle; testing occurred during the light phase. Water and food were supplied *ad libitum*.

### Apparatus

#### Rats

The arena was raised 86 cm above the ground, centered within a 2.5 m diameter circle of gray curtains to block extra-maze cues. Suspended 64 cm above the arena was a 1m-wide circular array of eight 45 W spotlights. At the center of the lights was a video camera. Arenas (rectangular: 120 ×60 cm; square: 85 ×85 cm; height 50 cm) were constructed from black medium-density fiberboard. The features were 0.5 cm thick polyurethane panels, in alternating black and white stripes (thickness 10 cm). In each corner of the arena was a white ceramic cylindrical feeder (diameter 8 cm, height 4.2 cm). Chocolate chips were used as a reward. During disorientation, animals were placed inside a light-tight box.

#### Chicks

The testing arenas were identical to those used for the rats. The feeders were metallic and rectangular (7 ×9 ×5 cm). The arena was placed 68 cm above the floor, and was lit by a single 35 W bulb suspended 90 cm above the arena. The arena was placed at the center of a 2 m diameter circle of thick vinyl curtains. Mealworms (*Tenebrio molitor*) were used as reward, and the disorientation chamber was cylindrical (diameter 10 cm, height 13 cm) and lifted from above to release the chick.

### Design

Testing lasted two days, three trials per day, with an inter-trial interval of approximately 45 minutes. Each corner of each arena served as a goal at least once, and goals for the remaining two trials were chosen pseudo-randomly to balance the total number of times each corner served as the goal. No goals were repeated on consecutive trials, in order to minimize learning across trials (t < 1.1, p > 0.25). The animals’ release points were chosen randomly from the four arena walls. For the unrewarded probe phase of each trial, animals were allowed to explore the arena for 60 seconds. The time spent within 5 cm of each of the feeders was recorded.

### Procedures

#### Rats

One day before testing, rats were provided with some chocolate chips in their cages. Before the first trial of each day, animals received a familiarization trial during which they were given three minutes to explore the arena and eat one chocolate chip placed at its center. For each test trial, chocolate chips were added to one feeder. The animal was allowed to explore until it had eaten a piece of chocolate, at which point it was removed for 15 seconds, before being placed back in to the arena from the same starting point. The animal was allowed to explore the arena until it had eaten a second piece of chocolate. This was to discourage the use of an alternation strategy, documented in rats as a method of foraging^63^. The animal was then disorientated for 30 seconds by placing it in a dark, covered box and rotating the box. Disorientation involved clockwise and then anticlockwise rotations (at least 720° in each direction). During this time, the feeder containing chocolate was removed from the arena and replaced with an identical, but empty, feeder. The arena was cleaned with 15% ethanol and rotated 90° clockwise to counteract the use of possible uncontrolled extra-maze cues. The animal was placed back into the arena from a randomly selected wall and allowed to search for 60 seconds. Trials in which the rat was unresponsive (no feeder approaches) were omitted; if a rat was unresponsive for more than three of the six trials, data from that rat were omitted entirely^64^.

#### Chicks

After being moved from the incubator to its home cage, each chick was given 2–3 mealworms. For the next two days, chicks were individually taken to the testing room and placed in a plain square arena (devoid of informative landmarks or geometry) with all black walls and identical black feeders at the corners (for familiarization). An object, identical to the imprinting object, was fixed to the arena floor. On the first day, chicks ate mealworms from a feeder at the center of the arena, and then in a corner. On the second day, chicks found worms in one corner, before being disoriented. Chicks were then released from the center of a randomly chosen wall and given 60 seconds to search for mealworms.

Test trials were administered for two days following familiarization. Before the first trial of each day, the chick was given a 60-second familiarization period inside the testing arena with a feeder containing mealworms at the center. For the test trials, the chick was released from the center of a randomly chosen wall. It was allowed to explore until it had eaten a mealworm, removed from the arena, and disorientated by rotating clockwise and anticlockwise for 30 seconds. During this time, the feeder containing mealworms was removed from the arena and replaced with an identical, but empty, feeder. The arena was wiped and rotated 90° clockwise. The chick was placed back into the arena from the center of a randomly selected wall and allowed to search for 60 seconds. Trials in which the chick was unresponsive (no feeder approaches) or distressed (made two attempts to jump out of the arena) were omitted. If a chick was unresponsive or distressed for more than three of the six trials, then data from that chick were omitted entirely.

All husbandry and experimental procedures complied with European Legislation for the Protection of Animals used for Scientific Purposes (Directive 2010/63/EU). Experiments with rats were carried out in the Psychology Department of Durham University, in accordance with the U.K. Animals (Scientific Procedures) Act of 1986; all experiments were approved by the university internal review board on animal testing. Experiments with chicks were carried out in the Animal Cognition and Neuroscience Laboratory of the Center for Mind/Brain Sciences at the University of Trento and were previously authorized by the University of Trento’s Ethics Committee for the Experiments on Living Organisms, and by the Italian Ministry of Health (auth. num. 201/2013-B). All experiments were performed in accordance with relevant guidelines and regulations.

## Supplementary information

Supplementary Materials.

## Data Availability

The datasets generated during and/or analyzed during the current study are available from the corresponding author on reasonable request.
