# Supplementary material for: Distinct and combined responses to environmental geometry and features in a working-memory reorientation task in rats and chicks
Source: Sci Rep. 2020 May 5;10:7508. doi: 10.1038/s41598-020-64366-w (PMC7200675; doi:10.1038/s41598-020-64366-w)
Supplement: Supplementary file 1 — Supplementary Materials. [file 41598_2020_64366_MOESM1_ESM.docx]

Supplementary Materials

**Distinct and combined responses to environmental geometry and features in a working-memory reorientation task in rats and chicks**

**Sang Ah Lee^a^, Joseph M. Austen^b^, Valeria Anna Sovrano^c,d^, Giorgio Vallortigara^c,d^, Anthony McGregor^b^, Colin Lever^b^**

**^a^** *Department of Bio and Brain Engineering, Korea Advanced Institute of Science and Technology, Daejeon, Korea*

**^b^** *Department of Psychology, Durham University, Durham, UK*

**^c^** *Centre for Mind/Brain Sciences, University of Trento, Rovereto, Italy*

**^d^** *Department of Psychology and Cognitive Science, University of Trento, Rovereto, Italy*

Experiment 4.

In Experiments 2 and 3, chicks showed no sign of using the striped wall to help them navigate in the working memory reorientation task. To ensure that this failure was not due to an inability to use this featural cue at all, we separately observed six naïve chicks (the same age, sex, housing/rearing conditions, and testing environments as those in Experiments 1–3) in one session of a reference-memory task in the square arena from Experiment 2, but with two feeders, one placed in the center of the striped wall, the other at the center of the black wall on the opposite side of the arena (locations A and B in the figure below).

The basic procedures for the task familiarization and test were identical to the other experiments, except that the goal location stayed constant over the entire session. The testing session consisted of eight training trials (feedback provided) and a ninth unrewarded, 60-second probe trial. In each training trial, the chick was released from the center and allowed to freely explore the arena until it found the food in the target feeder. The chick was removed from the apparatus once it ate the worm; first choice was recorded for the eight training trials, and both the first choice and the amount of time (over 60 seconds) that the chick spent in the correct half of the square arena were recorded for the test phase.

Overall, we found that they quickly learned to approach the correct feeder 69% of the time, which was significantly above chance (t(5) = 2.67, p=0.04). There was no difference between the three chicks observed with the goal at location A and the three tested at location B (t(4)=0.88, p=0.44). Accuracy increases linearly across trials (F(1,4)=14.44, p=0.019).

For the ninth trial, the chicks were given 60 seconds to freely move about the arena, without any food reward. We found that they spent 91.9% of that time in the correct half of the arena, which is exceedingly above what is expected by chance, (t(5) = 8.06, p<0.001).
